# Editorial to the Special Issue “Biological and Pharmacological Activity of Plant Natural Compounds”

**DOI:** 10.3390/molecules26010063

**Published:** 2020-12-25

**Authors:** Raffaele Pezzani, Sara Vitalini

**Affiliations:** 1 Endocrinology Unit, Department of Medicine (DIMED), University of Padova, 35128 Padova, Italy; 2 Associazione Italiana per la Ricerca Oncologica di Base, 35128 Padova, Italy; 3 Department of Agricultural and Environmental Sciences, Milan State University, via G. Celoria 2, 20133 Milan, Italy

Plant natural products are a valuable source of compounds with a healthy potential effect on living organisms, including animals and humans. These natural compounds are commonly called phytochemicals, specifically indicating their origin from the plant kingdom. It is frequently reported that phytochemicals merge with phytotherapeutics, which are molecules with a keen effect on health. This Special Issue entitled “*Biological and Pharmacological Activity of Plant Natural Compounds*” focused its attention on such plant-derived products and aimed to expand our knowledge on bioactive effects in preclinical models.

This Special Issue consisted of 4 reviews and 11 research articles that substantially contributed to the mission of *Molecules,* i.e., to increase scientists’ and readers’ perspectives and knowledge on plant natural products. 

The first review evaluated *Tabebuia impetiginosa* (Mart. ex DC.) Mattos, an Amazonian plant traditionally used against fever, malaria, bacterial and fungal infections, and skin diseases [1]. The work emphasized that the main effect of this plant could derive from its anti-inflammatory activity together with the presence of immunomodulatory compounds. In addition, the authors suggested that even if the biological effects of *T. impetiginosa* are clearly detectable, future research needs to better characterize its mechanisms of action, which until now remain elusive.

The comprehensive work of De Carvalho and collaborators explored the phytochemical composition, biological and toxicological properties of four fruit species, i.e., *Talisia esculenta, Brosimum gaudichaudii, Genipa americana,* and *Bromelia antiacantha* [2]. They reported that these plants demonstrated anti-inflammatory, antitumor, and photosensitizing properties, in addition to providing key molecules with pharmacological and healthy activity.

Another review investigated the wide spectrum of biological activities of epigallocatechin gallate (EGCG), the main bioactive component of tea [3]. The mechanism of action along with signaling pathways and its pharmacological properties (antioxidative, anti-inflammatory, and antitumor) were reported, suggesting that EGCG could play a key role in the future treatment or prevention of cancer.

Interestingly, the work of Diniz and co-authors evaluated the antidepressant effects of cinnamic acids [4]. Such molecules, as reported in different animal model experiments of behavioral disorders, indicated their potential applicability as antidepressant drugs, given their low costs in certain cases. Cinnamic acids could have a future in the treatment of depression and other psychiatric conditions.

In addition to reviews, 11 preclinical studies were performed, ranging from the pharmacological effects to the biological impact on cell processes and/or signaling pathways.

*Mahonia aquifolium* (Pursh) Nutt. is a plant with potential anticancer effects. It has been studied in association with doxorubicin in lung cell models demonstrating stimulating antiproliferative results [5]. *M. aquifolium* extract showed apoptosis activation, cell cycle modulation, and a decrease in the invasion process. Furthermore, the synergistic effects of this plant extract and doxorubicin (with the subsequent decrease in toxic effects) suggested its potential application in higher models. 

Seeds of *Pharbitis nil* (L.) Choisy, a traditional plant used in East Asia to treat inflammation and cancer, were used in cell models of colorectal cancer to evaluate the antiproliferative effects of the purified extract [6]. Cell cycle modulation and apoptosis induction were observed, with changes in the RAS/ERK and AKT/mTOR pathways. In addition, the extract preserved muscle cell function, suggesting the seeds of *P. nil* as a promising novel nature-derived drug against colorectal cancer.

Four different secoiridoids were studied for their potential anticancer effects on human epidermoid carcinoma (A431) and non-small cell lung cancer (A549) cell lines [7]. One of these compounds, multifloroside showed the highest inhibitory activity against A431 cells, with the ability to suppress colony formation, induce S cell cycle arrest, and increase reactive oxygen species (ROS) production and mitochondrial membrane potential. 

A work on the phenolic compounds from 18 different *Hibiscus acetosella* accessions were assessed for their antibacterial activity and antioxidant activity (associated with the level of anthocyanins) [8]. For the first time, the antibacterial activity of *H. acetosella* was shown on the Gram-negative *Pseudomonas aeruginosa* and on the Gram-positive *Staphylococcus aureus,* reserving an antimicrobial perspective in higher organisms for these compounds.

Another work evaluated the influence of maltodextrin and inulin (used as a carrier) on the quantitative and qualitative composition of the polyphenolic profile of *Amelanchier alnifolia* Nutt. fruit, juice, and pomace powders, in order to study the polyphenol profile and antioxidant properties [9]. The results showed the strong effect of the processing and matrix composition on the preservation of the antioxidant properties of *A. alnifolia*. The authors claimed that properly designed high-quality powders are necessary to obtain valuable nutraceutical additives for future use in humans.

*Ageratina havanensis* (Kunth) R. M. King and H. Robinson was used as a basis to compare the quantitative chemical composition of extracts in its flowering and vegetative stages [10]. This plant, typical to the Caribbean and Texas, was also studied for its antioxidant activity and to evaluate its effects on P-glycoprotein (P-gp) function. The results showed the content of major flavonoids of *A. havanensis*, its antioxidant effects, and the ability to inhibit P-gp. Thus, this plant species is a source of new potential inhibitors for drug efflux.

In an animal study, Wali and colleagues showed the protective effect of zingerone (ZIN) against lipopolysaccharide-induced oxidative stress, DNA damage, and cytokine storm [11]. They reported the strong antioxidative effect of ZIN, with the restoration of plasma enzymes and attenuated plasma proinflammatory cytokines and sepsis biomarkers. Thus, ZIN appears to be a convincing candidate for validation in future clinical trials for its anti-inflammatory and antioxidant properties. 

Similar to ZIN, juglone (5-hydroxyl-1,4-naphthoquinone), a well known black walnut (*Juglans nigra*) derivative, was evaluated as an anti-inflammatory compound in a mouse macrophage cell model (J774.1 cells) [12]. The results showed that juglone could reduce inflammatory cytokine production and NLRP3 inflammasome activation in macrophages, indicating this molecule to be a possible therapeutic tool to control inflammation.

Another potential anti-inflammatory compound was nootkatone (NTK), a sesquiterpenoid found in the essential oils of many species of the *Citrus* genus, tested in mice models of acute and chronic inflammation [13]. The authors reported that NTK was effective in reducing IL1-β and TNF-α production, COX-2 activity, and that it could antagonize the H1 receptor in acute assays. However, further studies are necessary to understand its mechanism of action in chronic inflammation.

Furthermore, renal diseases were investigated in in vivo models. Hlophe et al. analyzed the effects of a plant-derived lanosteryl triterpene, known as RA-3 [14]. The compound was able to reduce blood urea nitrogen, creatinine, uric acid, and xanthine oxidase biomarkers in rats, suggesting antihyperuricemic and nephroprotective properties. Moreover, antioxidant status with a decrease in malondialdehyde content was observed, again indicating the potential role of RA-3 in renal diseases.

Hesperidin (HSP), one of the principal bioflavonoids of *Citrus* fruits, was extracted from orange bagasse and tested to eliminate dark eye circles in in vitro artificial 3D skin [15]. The most effective methods for HSP nanonization were explored, and this nanonized compound was found to be the most skin-friendly and could be potentially used in cosmetics.

Overall, this Special Issue significantly contributes to stressing the importance of the biological and pharmacological activities of plant natural compounds. The aforementioned preclinical results revealed novel molecules with exciting properties that could potentially be useful for further human studies, for nutritional purpose, and for therapeutic aid. 
